# The Association Between Gonarthrosis Pain Severity and Radiographic Findings on X-Ray: A Cross-Sectional Study

**DOI:** 10.7759/cureus.35258

**Published:** 2023-02-21

**Authors:** Abdullah A Altuwairqi, Huda M Qronfla, Lama S Aljehani, Khalid G Khashoggi

**Affiliations:** 1 Department of Orthopedic Surgery, King Abdulaziz University Hospital, Jeddah, SAU; 2 College of Medicine, King Abdulaziz University Hospital, Jeddah, SAU; 3 Department of Radiology, King Abdulaziz University Hospital, Jeddah, SAU

**Keywords:** radiograph, womac, kellgren-lawrence, pain, osteoarthritis, knee osteoarthritis

## Abstract

Introduction: Knee osteoarthritis (KOA) is a degenerative joint disease that progresses over time due to articular cartilage loss. Orthopedic surgeons use plain radiography (X-ray) with an anteroposterior (AP) standing (weight-bearing) view, which is currently considered the gold standard modality, to diagnose KOA. They base this diagnosis on the clinical history and physical examination of the knee joint. However, many previous studies have reported a weak correlation between knee-joint structural abnormalities on X-rays and described pain. Therefore, our study aimed to assess the incompatibility between patients’ pain-severity complaints and radiographic findings on standing AP view. No similar study has been recently published in the Middle East.

Methods: 158 participants were selected for the study from King Abdulaziz University Hospital, Jeddah, between March 2022 and August 2022. We graded the patients’ AP knee radiographs using the Kellgren-Lawrence (KL) grading scale and the Western Ontario and McMaster Universities Osteoarthritis (WOMAC) index, by which we made phone calls to assess pain severity on a 0-10 pain subscale.

Results: We found a significant association between the 0-10 pain subscale and WOMAC questions describing difficulty in sitting (*p *< 0.05). Comparing KL scores on X-ray on a 0-10 pain subscale, we found a significant association between mild pain and severe radiological findings on X-ray and vice versa. In addition, the comparison between KL scores on X-ray and WOMAC questions describing difficulty in sitting showed a significant association between moderate difficulty in sitting and severe radiological findings and vice versa.

Conclusion: Our results indicated that there was a significant relationship between mild pain and severe radiological findings on X-rays and vice versa. Additionally, there was a significant relationship, based on the KL score and the WOMAC item that assessed sitting difficulty, between moderate sitting difficulty and severe radiological findings and vice versa. This may suggest that central and peripheral sensitization could be one factor in the causes of pain.

## Introduction

Knee osteoarthritis (KOA) is a degenerative joint disease that progresses over time owing to articular cartilage loss. Typically, KOA has two types: primary osteoarthritis (OA), without apparent underlying causes, and secondary OA, resulting from either abnormal articular cartilage, such as rheumatoid arthritis or from abnormal force to the knee joint (in cases of trauma) [1]. During clinical visits, patients with KOA usually exhibit knee pain, morning stiffness, crepitations, and movement limitations [2]. In addition to clinical history and physical examination of the knee joint, orthopedic physicians use plain radiography (X-ray) with an anteroposterior (AP) standing (weight-bearing) view, which is currently considered the gold standard modality for diagnosing OA. They also take an X-ray with a lateral and skyline view [3]. Despite common manifestations of symptomatic KOA, their association with knee pain is highly variable, with different intensities and severities that worsen over time and during different daily activities [1,2]. 

Many previous studies have reported a weak correlation between structural knee joint abnormalities on X-rays and reported pain [4]. Furthermore, various studies have examined the association between knee pain severity among patients with KOA and radiological findings using X-ray and the Kellgren-Lawrence (KL) grading scale. More recently, a cohort study of 2,322 participants with or at risk for KOA was conducted in Toronto, Canada. Researchers examined the relationship between knee pain patterns, pain severity, severity, and duration of radiographic findings of OA. To assess patients’ knee pain, they used the Intermittent and Constant Osteoarthritis Pain (ICOAP) score, the Western Ontario and McMaster Universities Osteoarthritis Index (WOMAC) pain subscale, and the visual analog scale (VAS). They used the KL grade to categorize the severity of radiographic KOA. The results showed that knee pain patterns were associated with the stage and duration of radiographic disease and pain severity [5]. 

Another prospective study in Hong Kong in 2014 was conducted on 193 patients with primary KOA. The study aimed to determine whether there was a link between ultrasonographic findings and pain score and whether these findings showed a better association with pain level than conventional X-rays. The findings revealed that both imaging modalities had a significant relationship with pain aspects; neither was superior to the other but rather complementary [6]. Another prospective study was conducted in the same year in Turkey, involving 117 female patients with KOA, to assess the link between radiological findings and functional status. Their results revealed that functional status and knee muscle strength in patients with KOA were similar in those with and without radiological changes [7].

Moreover, in Turkey in 2012, a cross-sectional study was conducted on 114 patients with KOA to explore the relationship between pain, disability, and radiographic findings. The results revealed that the KL grading scale was positively and significantly associated with age and disease duration. However, the difference in pain severity, disability, or stiffness according to the KL grading scale was not statistically significant. Therefore, the study found no correlation between function and radiographic features [8]. A prospective study in Canada evaluated the relationship between radiological severity and clinical and psychological factors in 100 patients with KOA. It found that advanced radiological OA and knee pain were significantly linked to disability, in addition to other psychological consequences, including depression and social isolation [9].

Therefore, in this study, we aimed to assess the relationship between the severity of KOA pain among Saudi patients and radiographic findings at King Abdulaziz University Hospital (KAUH) during the previous six months, between March 2022 and August 2022. In addition, we aimed to assess the incompatibility between patients’ pain-severity complaints and radiographic findings on standing AP view, aiming to update the old data from previous studies [6-9]. To date, no similar study has been conducted in the Middle East. Finding a strong relationship between a specific change in radiographs and pain severity might lead to a better understanding and treatment of the causes of pain in these patients.

## Materials and methods

We conducted this cross-sectional study at KAUH, Jeddah, Saudi Arabia, between March 2022 and August 2022. The participants in our study were patients with KOA who had undergone a standing anteroposterior (AP) view X-ray of the knee. Saudi adults of all ages who had data of standing AP view radiographs of the knee through the orthopedic department were included in our study. However, we excluded patients who were surgically treated with total/hemi knee replacement (arthroplasty). The estimated required sample size was 248 participants with a 95% confidence level and a margin of error of 5%. We performed our calculations using the Raosoft sample size calculator. 

All patient identifiers were removed for data collection, database storage, and statistical analysis. The patients’ records were assigned numbers using a numbering reference sheet and referred to during communication and study interactions using that reference number. Individual participants’ information cannot be identified in any publications or presentations resulting from this study. Only aggregated data were provided and analyzed. Data were stored in a secure computer system in the principal investigator’s office, accessible only by the researchers during the study. 

We recruited the participants through the hospital’s information system. We evaluated the participants’ pain severity by means of structured phone interviews because the pain severity scoring system is not united in the hospital’s information system. The KL score, which describes joint changes in severity on AP standing X-ray, has five stages starting from 0 to 4, which means no osteoarthritic changes, mild, moderate, and severe, respectively. Demographic data (file number, phone number, age, sex, and nationality) were recorded electronically on organized Microsoft Excel sheets (Redmond, USA). During the phone call, we asked structured questions of the participants, which assessed pain severity using a scale of 0 to 10, and we asked four extracted questions from the WOMAC pain subscale. We entered and analyzed the data using IBM Corp. Released 2019. IBM SPSS Statistics for Windows, Version 26.0. Armonk, NY: IBM Corp. We compared three groups (mild, moderate, and severe) using a one-way ANOVA test and used an independent t-test to compare two independent variables (male and female). Ethical approval was provided by the Research Ethics Committee at King Abdulaziz University, Faculty of Medicine, with the registration number HA-02-J-008.

## Results

Our study aimed to evaluate the relationship between KOA pain severity and radiographic findings and to assess the incompatibility between severity complaints and radiographic findings on standing AP view among Saudi patients at KAUH.

Two hundred nineteen patients, all of whom were Saudi, visited our clinic and were diagnosed with KOA; their data were considered for our analysis. However, only 153 of the patients were willing to participate in our study. The ages of the patients with KOA ranged from 30 to 84 years, with a mean of 59.2. The majority of the patients were female: 128 (83.7%); 25 were male (16.3%). 

All patients had already been diagnosed with KOA and had been given a standing AP view X-ray of the knee in the previous six months. We reviewed and scored the radiographic findings using the KL score, showing that 27 (17.6%) of the patients had mild changes, 54 (35.3%) had moderate changes, and 72 (47.1%) had severe changes. This indicated that most of the patients were categorized as having KOA pain ranging from moderate to severe. 

Furthermore, we asked the patients about their pain severity using the WOMAC pain subscale and 0-10 pain scale, where 0 = no pain, 1 and 2 = very weak pain, 3 and 4 = weak pain, 5 = moderate pain, 6 and 7 = strong pain, 8 and 9 = very strong pain, and 10 = intense pain. Our analysis showed that the majority of the patients reported intense pain, 120 (78.4%), whereas a few of them reported very weak to moderate pain, 31 (20.3%). In addition, only two (1.3%) patients had no pain at all. When the patients responded to the WOMAC, 58 (37.9%) had experienced extreme pain at night while in bed, 28 (18.3%) had severe pain, 17 (11.1%) had moderate pain, six (3.9 %) had mild pain, and 44 (28.8%) had none.

Regarding standing difficulty, 72 (47.1%) patients reported extreme difficulty, 32 (20.9%) had severe difficulty, 23 (15%) had moderate difficulty, 15 (9.8%) had mild difficulty, and 11 (7.2%) had none. Furthermore, 43 (28.1%) patients reported extreme difficulty in sitting, 33 (21.6%) severe, 35 (22.9%) moderate, 19 (12.4%) mild, and 23 (15%) none. Asked about the difficulty in rising from sitting, 77 (50.3%) patients reported extreme difficulty, 30 (19.6%) reported severe difficulty, 26 (17%) reported moderate difficulty, nine (5.6%) reported mild difficulty, and 11 (7.2%) reported none. 

We compared the KL score on X-ray to the 0-10 pain subscale and WOMAC subscore. We concluded, based on the results of the p-values, that there was a significant difference between the 0-10 pain subscale and the third WOMAC question, which was about the degree of difficulty in sitting (p <0.05) (Table 1).

**Table 1:** This table shows a comparison between categorical groups within the pain severity (0-10) subscale and WOMAC question that describes the difficulty in sitting by testing differences of means using variance showing significant associations (p-value < 0.05) WOMAC: Western Ontario and McMaster Universities Osteoarthritis Index

ANOVA						
		Sum of Squares	df	Mean Square	F	Sig.
Pain severity (0 - 10)	Between Groups	14.130	2	7.065	3.353	0.038
	Within Groups	316.079	150	2.107		
	Total	330.209	152			
Question 3: What degree of difficulty do you have in sitting?	Between Groups	20.775	2	10.387	5.642	0.004
	Within Groups	276.167	150	1.841		
	Total	296.941	152			

Other variables, such as other WOMAC questions regarding pain at night, difficulty in standing, and difficulty in rising from sitting, showed no significant differences (p > 0.05). While comparing KL scores on X-ray to the 0-10 pain subscale, there was a significant association between mild pain and severe radiological findings on X-ray and vice versa (Table 2). Furthermore, the comparison between KL scores on X-ray and the WOMAC question that assessed difficulty in sitting showed a significant association between moderate difficulty in sitting and severe radiological findings, and vice versa (Table 2).

**Table 2:** Comparison between pain severity (0-10 pain subscale and WOMAC) and KL grades on X-ray. HSD: Honestly significant difference; KL: Kellgren-Lawrence scale; Std.: standard; WOMAC: Western Ontario and McMaster Universities Osteoarthritis Index

Multiple Comparisons							
Tukey HSD							
	(I) Radiological findings on x-ray KL Score	(J) Radiological findings on X-ray KL Score	Mean Difference (I-J)	Std. Error	Sig.	95% Confidence Interval	
						Lower Bound	Upper Bound
Pain severity (0 - 10)	Mild Between Groups	Moderate	-0.648	0.342	0.144	-1.46	0.16
		severe	-0.847	0.328	0.029	-1.62	-0.07
	Moderate Total	Mild	0.648	0.342	0.144	-0.16	1.46
		severe	-0.199	0.261	0.727	-0.82	0.42
	severe	Mild	0.847	0.328	0.029	0.07	1.62
		Moderate	0.199	0.261	0.727	-0.42	0.82
Question 3: What degree of difficulty do you have in sitting?	Mild	Moderate	0.352	0.320	0.515	-0.41	1.11
		severe	-0.463	0.306	0.288	-1.19	0.26
	Moderate	Mild	-0.352	0.320	0.515	-1.11	0.41
		severe	-0.815	0.244	0.003	-1.39	-0.24
	severe	Mild	0.463	0.306	0.288	-0.26	1.19
		Moderate	0.815	0.244	0.003	0.24	1.39

Comparing gender with pain severity, our data showed significant differences (p < 0.05) in the 0-10 pain subscale and WOMAC questions 1, 2, and 4. Forty-four (34.4%) female patients experienced intense pain, while only two (1.6%) had no pain, and the other two (1.6%) had very weak pain. In contrast, eight (32%) male patients reported strong pain, while none were pain-free (0%). In the WOMAC question that assessed pain severity at night while in bed and asked if pain disturbed sleep, 56 (43.8%) female patients reported pain during sleep, while five (3.9%) had mild pain. Sixteen (64%) male patients had no pain during sleep, and one (4%) patient had mild pain. In the WOMAC question that assessed difficulty in standing, 64 (50%) female patients experienced extreme pain, whereas only nine (7%) had no pain. In contrast, eight of the male patients (32%) had extreme pain, and two (8%) had no pain. In the WOMAC question, which assessed difficulty in rising from sitting, 69 (53.9%) female patients exhibited extreme pain, whereas only six (4.7%) had mild pain. Similarly, most of the male patients (32%) had extreme pain, while only three (12%) had mild pain; the other three (12%) patients had no pain.

## Discussion

In this cross-sectional study, we evaluated whether there was a relationship between KOA pain severity and radiographic findings and assessed the incompatibility between patient severity complaints and radiographic findings on standing AP view among Saudi patients at KAUH. Our results demonstrated a significant association between mild pain and severe radiological findings on radiography and vice versa in comparison with the KL score on X-ray to the 0-10 pain subscale. In addition, the comparison between KL scores on X-ray and the WOMAC question that assessed difficulty in sitting showed a significant association between moderate difficulty in sitting and severe radiological findings, and vice versa.

KOA is a common disease with a high prevalence worldwide [10], especially among female patients. This is explained by the female sex hormone estrogen, which plays a role in the pathophysiology of degenerative diseases of the musculoskeletal [11]. 

A previously published cross-sectional study illustrated that the majority of participants were female patients [8]. Similarly, in our study, 83.7% of the participants were female patients. This imbalance could be explained by hormonal variations between male and female patients in relation to interleukin-1, which plays a role in OA. It has been reported that in postmenopausal women, as estrogen levels decrease, the levels of interleukin-1 decrease, leading to OA [12]. In addition, another study was conducted to determine sex differences in correlations between symptoms and radiographic severity in patients with KOA. The study reported that women in all KL grades had more severe symptoms than men by using the WOMAC pain subscale [13]. In addition, women’s OA progression could be explained by the estrogen deficiency occurring after menopause. Therefore, some studies have suggested estrogen replacement therapy after menopause to protect against the development of knee osteoarthritis [12].

Another published article showed that 32% of patients had mild KOA, 33% had moderate KOA, and 23% had severe KOA on X-ray [14]. However, in our study, we found that the majority of our patients had moderate-to-severe osteoarthritic changes in the standing AP view of knee radiographs. Compared with Turkey, there is a higher incidence of physical inactivity among the Saudi population, which could be up to 85% among male and 91% among female patients [14]. Hence, this may affect the prognosis of the disease because exercise plays a role in pain management [15].

A longitudinal cohort study was conducted to measure KOA pain severity and analyze the radiographic findings with duration and severity separately [5]. It found that approximately half of the patients had mild-to-moderate knee pain based on the VAS scores. Furthermore, most of them had moderate-to-severe KL scores after excluding KL 0 and 1 [5]. In contrast, in our study, we performed an analysis to assess radiographic severity through KL scores in relation to the 0-10 pain subscale. We found that there was a significant association between mild pain and severe radiological findings on radiography and vice versa. This discordance could be better explained by peripheral or central sensitization in patients with KOA, particularly in those with severe pain complaints but a mild KL score [16,17].

A study conducted in Turkey could not establish an association between WOMAC scores and the KL grading scale [8]. In this study, we assessed whether there was any relationship between radiographic findings, pain, and disability in patients with KOA. We found that there was a significant association between one of the WOMAC disability questions regarding the difficulty in sitting and radiological severity. Our findings also demonstrated that patients with moderate difficulty in sitting had severe osteoarthritic changes. Moreover, the patients with severe difficulty in sitting had moderate osteoarthritic changes in radiographs according to the KL score. This could suggest that pain or other disease-related parameters may not be utterly dependent on structural damage, such as cartilage loss, which is typically recognized on plain radiographs as a reduction in joint space and an important structural feature. However, the cartilage is not innervated and consequently cannot be considered a direct source of pain in patients with mild-to-moderate disease. Furthermore, the phenomenon known as peripheral and central sensitization may explain why some people with KOA experience severe pain regardless of the level of structural damage [17].

In our study, we had a limited number of blinded readers for X-rays, which decreased the possibility of bias and had more accuracy in reading and scoring. In addition to online surveillance, we collected our data using archival data, in addition to questions via phone. 

However, our study has some limitations. First, the sample size was small and from a single center. Therefore, our results cannot be generalized to the whole population. Furthermore, we did not assess obesity or other comorbidities that could play a role in the physical functional status of patients with KOA [12]. 

## Conclusions

Our results showed multiple significant values, which we interpreted as revealing that the patients with mild knee pain showed severe radiological findings on X-ray and vice versa, and also that patients with moderate knee pain showed severe radiological findings on X-ray and vice versa. Therefore, we recommend that orthopedic surgeons identify the risk factors for KOA and develop a screening program in coordination with radiologists for early detection and management to improve the functional status in patients and prevent the transition from mild to severe stages of KOA. Additionally, to fully understand the relationship between radiological damage and functional status in patients with KOA, we recommend further studies with larger samples and further assessment of risk factors that could have an impact on KOA development, such as obesity, vitamin D deficiency, race, dietary factors, and occupation.
